# Effect of Acute, Subacute, and Repeated Exposure to High Altitude (5050 m) on Psychomotor Vigilance

**DOI:** 10.3389/fphys.2018.00677

**Published:** 2018-06-04

**Authors:** Matiram Pun, Sara E. Hartmann, Michael Furian, Adrienna M. Dyck, Lara Muralt, Mona Lichtblau, Patrick R. Bader, Jean M. Rawling, Silvia Ulrich, Konrad E. Bloch, Marc J. Poulin

**Affiliations:** ^1^Department of Physiology and Pharmacology, Cumming School of Medicine, University of Calgary, Calgary, AB, Canada; ^2^Hotchkiss Brain Institute, Cumming School of Medicine, University of Calgary, Calgary, AB, Canada; ^3^Pulmonary Division, Sleep Disorders Centre and Pulmonary Hypertension Clinic, University Hospital Zurich, Zurich, Switzerland; ^4^Faculty of Kinesiology, University of Calgary, Calgary, AB, Canada; ^5^Department of Family Medicine, Cumming School of Medicine, University of Calgary, Calgary, AB, Canada; ^6^Libin Cardiovascular Institute of Alberta, Cumming School of Medicine, University of Calgary, Calgary, AB, Canada; ^7^O’Brien Institute for Public Health, Cumming School of Medicine, University of Calgary, Calgary, AB, Canada; ^8^Department of Clinical Neurosciences, Cumming School of Medicine, University of Calgary, Calgary, AB, Canada

**Keywords:** altitude, psychomotor vigilance task, actigraphy, sleep, hypoxia, brain

## Abstract

**Aim:** High altitude (HA) hypoxia may affect cognitive performance and sleep quality. Further, vigilance is reduced following sleep deprivation. We investigated the effect on vigilance, actigraphic sleep indices, and their relationships with acute mountain sickness (AMS) during very HA exposure, acclimatization, and re-exposure.

**Methods:** A total of 21 healthy altitude-naive individuals (25 ± 4 years; 13 females) completed 2 cycles of altitude exposure separated by 7 days at low altitude (LA, 520 m). Participants slept at 2900 m and spent the day at HA, (5050 m). We report acute altitude exposure on Day 1 (LA vs. HA1) and after 6 days of acclimatization (HA1 vs. HA6). Vigilance was quantified by reaction speed in the 10-min psychomotor vigilance test reaction speed (PVT-RS). AMS was evaluated using the Environmental Symptoms Questionnaire Cerebral Score (AMS-C score). Nocturnal rest/activity was recorded to estimate sleep duration using actigraphy.

**Results:** In *Cycle 1*, PVT-RS was slower at HA1 compared to LA (4.1 ± 0.8 vs. 4.5 ± 0.6 s^-1^, respectively, *p* = 0.029), but not at HA6 (4.6 ± 0.7; *p* > 0.05). In *Cycle 2*, PVT-RS at HA1 (4.6 ± 0.7) and HA6 (4.8 ± 0.6) were not different from LA (4.8 ± 0.6, *p* > 0.05) and significantly greater than corresponding values in *Cycle 1*. In both cycles, AMS scores were higher at HA1 than at LA and HA6 (*p* < 0.05). Estimated sleep durations (TST) at LA, 1st and 5th nights were 431.3 ± 28.7, 418.1 ± 48.6, and 379.7 ± 51.4 min, respectively, in *Cycle 1* and they were significantly reduced during acclimatization exposures (LA vs. 1st night, *p* > 0.05; LA vs. 5th night, *p* = 0.012; and 1st vs. 5th night, *p* = 0.054). LA, 1st and 5th nights TST in *Cycle 2* were 477.5 ± 96.9, 430.9 ± 34, and 341.4 ± 32.2, respectively, and we observed similar deteriorations in TST as in *Cycle 1* (LA vs. 1st night, *p* > 0.05; LA vs. 5th night, *p* = 0.001; and 1st vs. 5th night, *p* < 0.0001). At HA1, subjects who reported higher AMS-C scores exhibited slower PVT-RS (*r* = -0.56; *p* < 0.01). Subjects with higher AMS-C scores took longer time to react to the stimuli during acute exposure (*r* = 0.62, *p* < 0.01) during HA1 of *Cycle 1*.

**Conclusion:** Acute exposure to HA reduces the PVT-RS. Altitude acclimatization over 6 days recovers the reaction speed and prevents impairments during subsequent altitude re-exposure after 1 week spent near sea level. However, acclimatization does not lead to improvement in total sleep time during acute and subacute exposures.

## Introduction

High altitude (HA) exposure is associated with low blood oxygen saturation due to the decreased partial pressure of oxygen as a result of reduced barometric pressure (West, 1996). Compared to slow acclimatizing trekking, rapid ascent to HA (via motor vehicle or air travel, for example) leads to profound hypoxemia and related pathophysiological consequences (Murdoch, 1995; West, 2008; Bloch et al., 2009; Strapazzon et al., 2015). The physiological responses to HA exposure include heart rate elevation, increased ventilation, and diuresis and fatigue on exertion (Nussbaumer-Ochsner and Bloch, 2007; Richalet et al., 2012; Luks, 2015). Failure to acclimatize due to rapid ascent leads to acute altitude illness such as acute mountain sickness (AMS) (Hackett and Roach, 2001; Bartsch and Swenson, 2013; Luks et al., 2017). The continuous gain in elevation and prolonged exposure to altitude can lead to adverse neurological consequences (Basnyat et al., 2004; Wilson et al., 2009; Strapazzon et al., 2014; Phillips et al., 2017) and possibly impaired cognitive function although this has not been consistently demonstrated (Latshang et al., 2013; Roach et al., 2014; Beer et al., 2017).

Sleep disturbance is reported commonly during altitude exposure (Johnson et al., 2010; Latshang et al., 2013; Bloch et al., 2015) due to hypoxia and new environmental conditions such as cold, uncomfortable sleeping quarters, and disturbed sleep due to increased frequency of urination (Windsor and Rodway, 2012). Further, exposure to HA is associated with a higher incidence of periodic breathing (Bloch et al., 2010; Ainslie et al., 2013), a disturbance of ventilatory control commonly observed in HA sojourners. Periodic breathing is characterized by waxing and waning ventilation, punctuated with periods of hyperventilation and then central apneas or hypopneas. Affected individuals often suffer from frequent arousals and poor sleep (Nussbaumer-Ochsner et al., 2012).

Poor sleep at altitude may affect daytime performance, especially of those tasks that require sustained attention and delicate fine-motor dexterity. The speed of response to light stimuli in the psychomotor vigilance test (PVT), a validated indicator of alertness, is slower following sleep deprivation (Van Dongen and Dinges, 2005; Lim and Dinges, 2008). Several studies have reported negative effects of sleep-disordered breathing (Kim et al., 2007; Lee et al., 2011; Kitamura et al., 2017), sleep deprivation (Van Dongen and Dinges, 2005; Lim and Dinges, 2008), and HA exposure (Latshang et al., 2013; Roach et al., 2014) on psychomotor vigilance tasks. Hence, these conditions could have deleterious consequences for individuals at HA performing work that demands continuous attention, decision-making, delicate handling of tools, and heavy-duty machinery work.

The Atacama Large Millimeter/submillimeter Array (ALMA) Observatory at 5050 m above sea level (asl) in Chile provides a unique pattern of repeated high-altitude exposure. Scientists from all over the world visit the site periodically to conduct scientific observations, experiments, and data collection. Further, lowland-native Chileans work there in various capacities, including equipment maintenance, security operations, scientific data collection, and reporting. A normal commute schedule includes typically, flight to Calama (2900 m asl) from Santiago (520 m asl) and travel about 2 h by bus to the ALMA basecamp (2900 m asl). From there, for a week at a time they ascend to the ALMA Observatory. Workers then spend the day working at HA and return to basecamp by motor vehicle in the evening. These workers suffer from periodic hypoxia (∼6–8 h/day) at very HA, and then sleep in a hypoxic environment at HA. After a week of HA work, the workers descend to their altitude of residence near sea level to rest, before returning to work at HA for another cycle. This pattern repeats itself continuously. How this type of HA exposure affects immediate and long-term health, and consequently work performance, is unknown. Both poor sleep and hypobaric hypoxia at altitude may be related to impaired vigilant attention, thereby affecting daytime task performance.

To investigate the impact of very HA exposure on vigilant attention, we brought a group of young, healthy, altitude-naive individuals to the ALMA Observatory, Chile. We mimicked the pattern of hypoxia exposure that is experienced by workers, in order to study the acute and subacute effects of repeated very HA exposure over two work-shift cycles (i.e., approximately 4 weeks). We had three specific goals in this high-altitude research expedition. First, we wanted to study the effect of acute, subacute, and re-exposure to very HA on PVT reaction speed (PVT-RS) in healthy, young, altitude-naive individuals. Second, we wanted to assess relationships between PVT-RS, AMS-Cerebral (AMS-C) score, blood oxygen saturation and sleep indices as measured by actigraphy. Third, we wanted to explore whether acclimatization obtained in previous exposure is retained during subsequent exposures after 1 week of rest at low altitude (LA). We hypothesized that immediate exposure to very HA would be associated with a slower PVT-RS, worsened sleep indices and increased prevalence of AMS. We further hypothesized that these parameters would improve upon acclimatization, and would be less affected with repeated exposure to altitude.

## Materials and Methods

### Study Design and Setting

**FIGURE 1:**
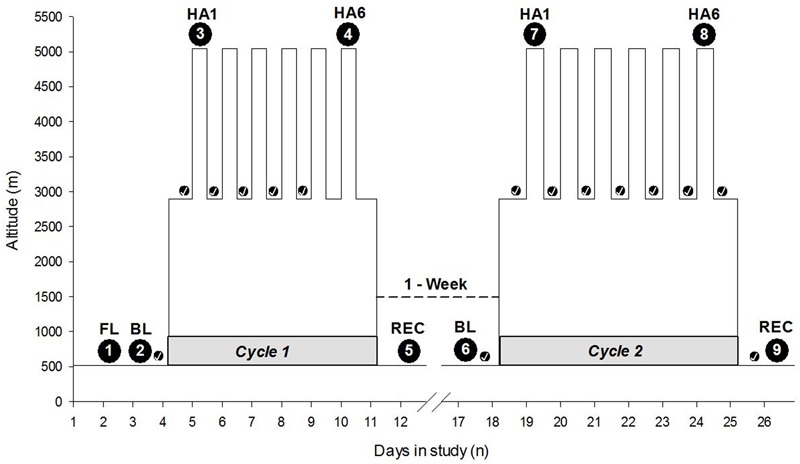
Expedition schedule and data collection time points. The *Y*-axis depicts altitude (meters), and the *X*-axis depicts the days of the entire expedition with two cycles at high altitude interspersed with 1 week at LA. The numbers within the large black circles depict time points of data collection. The small black circles with checkmark indicate the sleep nights with actigraphy, i.e., actigraphic data collection time points. The gray shaded horizontal part at the base of each cycle indicates *Cycle 1* and *Cycle 2*. “1-Week” with a dashed line connecting two cycles indicates time spent at LA. HA1, high altitude exposure at 5050 m asl on Day 1; HA6, high altitude exposure at 5050 m asl on Day 6 on the respective cycles of expedition; FL, familiarization; BL, baseline; REC, recovery; m, meters; n, numbers.

The study was carried out in Atacama Desert of northern Chile. Baseline measurements were taken in Santiago (520 m asl). After baseline measurements, the study participants traveled approximately 2 h by air followed by a 2-h bus to the ALMA Operation Support Facility (ASF; 2900 m asl), which serves as ALMA’s basecamp. Participants slept at the ASF for 7 nights and ascended by motor vehicle (∼45 min) to the AOS/ALMA to spend ∼7 h (range 4–8 h) per day at very HA. The ascent profile over 3 weeks from Santiago to the AOS has been illustrated in **Figure 1**. Altitude PVT measurements were taken at the ALMA Operation Site (AOS; 5050 m asl) located on the Chajnantor Plateau of the Atacama Desert. Actigraphy was performed over the span of a week cycle at 5050 and 2900 m sleep altitude, respectively. There were two 7-day cycles of HA exposure, separated by a break of 7 days at LA (Santiago, 520 m asl) (see schedule illustrated in **Figure 1**). The study participants’ high-altitude exposure schedule was designed to emulate that of workers at the ALMA Observatory.

### Participants

A total of 21 healthy altitude-naive individuals were recruited for the expedition. A total of 18 subjects were residents of Calgary and surrounding area (altitude 1100 m asl) in Canada and were screened for inclusion in the study at the Foothills Medical Center, Cumming School of Medicine, University of Calgary, Calgary, AB, Canada. Three subjects were from Zurich and surrounding area (altitude 490 m asl) in Switzerland and were screened at University Hospital of Zurich. Exclusion criteria included previous altitude intolerance < 3000 m, pregnancy and health impairment, which requires regular treatment. The screening criteria adhered to the ALMA HA Medical Examination guidelines. All participants provided written informed consent. The study was approved by the Conjoint Health Research Ethics Board of The University of Calgary (Ethics ID: REB 15-2709) and the Cantonal Ethics Committee of Zurich (2016-00048) and was registered at ClinicalTrials.gov (NCT02731456).

### Data Collection

The data collection was conducted over nine separate testing sessions before, during, and after two 7-day cycles at HA. The baseline and recovery measurements were taken at Santiago, Chile (520 m asl) while altitude data were collected at ALMA Observatory site (5050 m asl). Participants were familiarized with the measurements upon arrival in Santiago, whereas baseline measurements were performed on the day after. The objective of familiarization session was to make study participants familiar with experimental sessions, scoring sheets, instrumentation, and nature of invasiveness. The sleep data, acquired using actigraphy, were collected at the sleep altitudes (Santiago and the ASF) throughout the cycle, i.e., every night. The participants wore Actiwatches continuously throughout the expedition cycles unless otherwise due to technical and logistical reasons. The experimental sessions or specific data collection time points during HA exposure on the specific days have been illustrated in **Figure 1**.

### Psychomotor Vigilance Test and Trail Making Test

Since speed of psychomotor response to light stimuli is adversely affected following sleep deprivation (Sforza et al., 2004; Dorian et al., 2005; Basner and Dinges, 2011; Basner et al., 2011; Batool-Anwar et al., 2014), we utilized PVT-RS as a tool in assessing daytime function at altitude. Psychomotor vigilance was determined using a standard 10-min duration PVT assessment, previously described (Dorian et al., 2005; Drummond et al., 2005; Basner and Dinges, 2011). Time to react to randomly generated light stimuli was measured using a handheld Multiple Unprepared Reaction Time Test (MURT) device (Latshang et al., 2013). Several responses were obtained within a 10-min period, and the outcome was the mean reciprocal value of the RT (i.e., 1/RT). The PVT is a well-validated tool for vigilant attention deficits as a measure of neurobehavioral reaction speed due to sleep loss or deprivation (Basner and Dinges, 2011). The trail making tests (TMT) were administered in pen and paper version which contained circles containing numbers (Allen and Haderlie, 2010). The participants were instructed to connect circled numbers in sequence as quickly as possible without making an error. Whenever an error was made, the participants were asked to start again from the point where they made the error and continue. The times to complete the TMT were recorded in seconds.

### Sleep Monitoring: Actigraphy

Wrist actigraphy was recorded to estimate nocturnal rest as a surrogate of sleep (Latshang et al., 2016) (Actigraph 2.0, Minimitter; Philips Respironics, Murrysville, PA, United States). All participants wore an actimeter for a total of 9 days, including the baseline and recovery period at LA (520 m asl, Santiago) and 7 days at HA (2900 m asl, ALMA Operation Support Facility). The actigraphic data collected from the field were analyzed by the manufacturer’s guidelines and dedicated software (Respironics Actiware, Version 6.0.4). The data were exported and assessed by research team members experienced with Actigraphy. The variables analyzed from the actigraphy included total time in bed (i.e., time from lights-off to lights-on which each participant pressed marker switch in the Actiwatch and also recorded in the sleep diary), total estimated sleep time (TST; i.e., the sum of all epochs with activity below threshold), sleep efficiency (i.e., TST in percentage of time in bed), and sleep latency (i.e., time from lights-off to the beginning of the first three consecutive epochs with activity below the threshold) as described in the previous literature (Latshang et al., 2016). The definitions of sleep parameters such as awakenings and data analysis was carried out using the Actiware scoring algorithm as described previously (Drogos et al., 2016). The participants wore Actiwatches throughout the expedition.

### Acute Mountain Sickness Assessment and Vital Signs

Acute mountain sickness (AMS) was assessed using the Lake Louise Score (LLS) (Roach et al., 1993) and Environmental Symptom Questionnaire (ESQ) – Cerebral (AMS-C subscore) (Sampson et al., 1983). AMS diagnosis was made based on the LLS score of ≥ 5, and ESQ AMS-C weighted average score ≥ 0.7 (Maggiorini et al., 1998; Dellasanta et al., 2007; Nussbaumer-Ochsner et al., 2011). We have used AMS-C score for the analysis and interpretation. Handgrip (HG) strength was measured in the dominant hand using a strain-gauge dynamometer following manufacturer’s guideline (Lafayette, IN, United States). The HG peak strength has been reported in kilograms (kg). The resting arm blood pressure was recorded with automatic blood pressure monitor (Omron Healthcare, Inc., United States) in a comfortable sitting position. Blood oxygen saturation and heart rate were measured with a pulse oximetry.

### Data Analyses and Statistics

The data collected at different time points during the HA expedition have been expressed as the mean ± standard deviation (mean ± SD). The effects of altitude [baseline at sea level vs. HA exposure at Day 1 (i.e., acute exposure; HA1) and HA exposure at Day 6 (i.e., acclimatization; HA6)] were assessed with one-way repeated measures of analysis of variance (ANOVA) for each cycle independently. The sleep parameters were analyzed over different time points of sleep at HA (i.e., number of nights). In the second step of the analysis, three time-points (baseline, acute exposure, and acclimatization effects) were compared with repeated measures ANOVA to tease out acute (1st night sleep at HA) and acclimatization effects (fifth-night sleep) of altitude exposure in each cycle separately. Bivariate correlations were tested between variables of interest (reaction speed or changes in the reaction time with AMS score) using Pearson’s correlation coefficient. Multivariate regression analysis was performed to identify independent variables (SpO_2_, AMS-C score, and TST) associated with PVT-RS at HA1 and HA6 of both cycles to explore independent as well as interaction effects. The results were considered significant when the value of alpha was less than 0.05 (*p* < 0.05) after Bonferroni’s *post hoc* corrections, as appropriate. The recovery values were compared with baseline and HA values with two-tailed paired *t*-tests wherever necessary to explore the recovery status of the individuals. All the data analyses were carried out using the Statistical Package for the Social Sciences (SPSS), Version 24, IBM Corporation, United States.

## Results

A total of 21 healthy altitude-naive individuals aged, mean ± SD, 25.3 ± 3.8 years (8 males, 13 females) with body mass index of 22.8 ± 3.0 kg/m^2^ were recruited. The baseline characteristics such as weight (65.4 ± 9.3 kg), body mass index (22.7 ± 3.1 kg/m^2^), heart rate (65.5 ± 9.2 bpm), blood pressure (SBP = 114.2 ± 8.1 mmHg, DBP = 69.9 ± 6.2 mmHg, MAP = 84.7 ± 5.8 mmHg), peak HG strength (39.4 ± 13.1 kg) were taken at Santiago, Chile (520 m asl). The absoulte values from acute (HA1), acclimatization (HA6), and recovery (REC) sessions have been summarized in **Table 1**.

**Table 1:** Descriptive characteristics of the study participants.

Variables	*Cycle 1*				*Cycle 2*			
	Baseline SL	HA1	HA6	Recovery SL	Baseline SL	HA1	HA6	Recovery SL
Weight (kg)	65.4 ± 9.3	65.4 ± 9.1	64.6 ± 9.0	64.7 ± 9.3	64.9 ± 9.0	65.1 ± 9.0	64.3 ± 9.2	64.6 ± 9.1
Body mass index (kg/m^2^)	22.7 ± 3.1	22.7 ± 3.1	22.5 ± 2.9	22.5 ± 3.0	22.6 ± 3.0	22.6 ± 2.9	22.4 ± 3.0	22.4 ± 2.9
HR (bpm)	65.5 ± 9.2	87.5 ± 14.2	75.9 ± 12.4	61.8 ± 11.5	57.5 ± 10.6	79.8 ± 11.3	74.5 ± 9.3	62.9 ± 9.1
SBP (mmHg)	114.2 ± 8.1	114.7 ± 12.4	117.1 ± 8.2	107.0 ± 9.0	106.7 ± 9.4	111.3 ± 8.7	114.3 ± 10.0	103.6 ± 7.1
DBP (mmHg)	69.9 ± 6.2	72.2 ± 8.9	77.0 ± 8.9	65.7 ± 6.5	65.9 ± 7.3	73.7 ± 7.2	76.2 ± 6.4	62.5 ± 7.4
MAP (mmHg)	84.7 ± 5.8	86.3 ± 9.4	90.3 ± 7.5	79.4 ± 6.9	79.5 ± 7.5	86.2 ± 7.1	88.9 ± 6.6	76.2 ± 6.7
HG peak strength (kg)	39.4 ± 13.1	36.7 ± 10.4^a^	38.8 ± 12.7	39.9 ± 12.2^a^	38.0 ± 12.2	36.5 ± 13.9	39.2 ± 12.2	39.9 ± 13.0

*The descriptive characteristics of the 21 altitude-naive healthy individuals, who joined the high altitude expedition, born and raised at an altitude of ≤ 1100 m asl. Values are means ± SD. SL, sea level; HA1, high altitude at day 1; HA6, high altitude day 6; SBP, systolic blood pressure; DBP, diastolic blood pressure; MAP, mean arterial blood pressure; HR, heart rate; mmHg, millimeter of mercury; bpm, beats per minute; HG, handgrip; kg, kilogram; SD, standard deviation; kg, kilogram; m, meter. ^*a*^*n* = 20, Sex: Males (8) and Females (13) and average age: 25.3 ± 3.8 years.*

**FIGURE 2:**
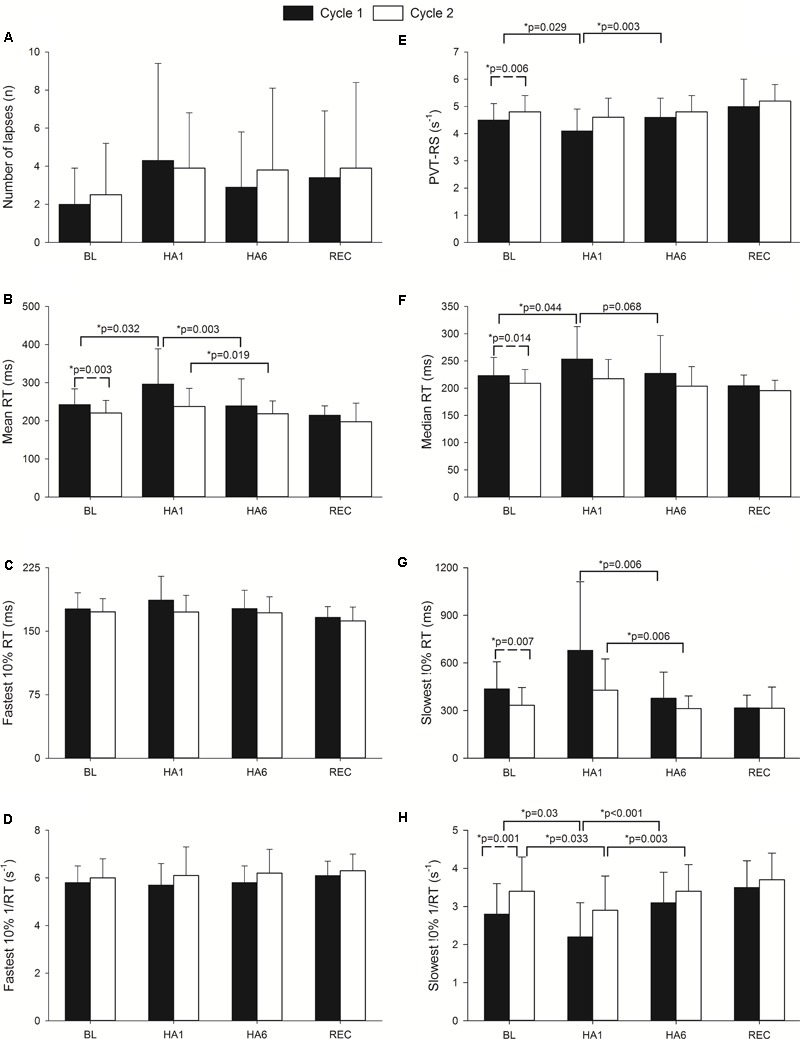
Effect of altitude exposure on PVT parameters during acute and acclimatization exposures. It illustrates changes in different PVT parameters during high altitude exposure. Four important time points of the expedition are illustrated (BL, baseline at LA; HA1, acute high altitude exposure; HA6, acclimatization exposure of both cycles; REC, recovery). The *Y*-axis depicts sleep parameters with the mean and standard deviation (Mean ± SD) of the PVT parameters. The *X*-axis depicts different expedition time points (BL1, HA1, HA6, and REC of *Cycle 1* and *Cycle 2*). Solid bars represent *Cycle 1* exposure while empty bars represent *Cycle 2* exposure. Here, panel **A** illustrates number of errors/lapses while panel **E** represents PVT reaction speed. Panels **B** and **F** represent mean and median of reaction times respectively. Panels **C** and **G** show fastest and slowest 10% reaction times while **D** and **H** depict reciprocals of them in respective orders. The significance shown with dashed line indicates baseline comparisons of two cycles. The comparison with solid lines is between different time points of respective cycles. n, number; RT, reaction time; ms, milliseconds; s^-1^, per second; PVT-RS, psychomotor vigilance test reaction speed; p, level of significance; *p*,^∗^ level of significance value with less than 0.05.

The sustained attention test (i.e., PVT) assessed by the MURT test device has been illustrated in **Figure 2**. The number of mistakes/lapses was not statistically significant during HA exposure although there was a trend to increase with acute exposure in both cycles. The individuals took a longer time to react (increase in mean and median reaction times) at HA during acute exposure (HA1), and they improved during acclimatization visit (HA6) in *Cycle 1*, but they were not significantly prolonged in *Cycle 2* compared to their respective baselines. The PVT-RS was impaired during acute exposure but improved in subsequent exposure. The PVT-RS was unaffected during *Cycle 2* (i.e., individuals were acclimatized in *Cycle 1*, as shown with values at HA6 and retained it during *Cycle 2*). The fastest 10% reaction times (i.e., optimum response times) did not significantly change at HA in both cycles but the slowest 10% reaction times (i.e., response times in the lapse domain) increased during acute exposure (HA1) and improved during acclimatization visits (HA6) in both cycles. The PVT parameters from baseline, acute exposure (HA1), acclimatization (HA6), and recovery (REC) have been illustrated in **Figure 2**. We also incorporated the trail-making test (TMT) which did not get worse during acute exposures (HA1) but improved during the acclimatization exposures (HA6) compared to baseline in both *Cycles*. There was a significantly higher incidence of AMS during the first exposure but not during the acclimatization exposure with both scoring criteria of AMS [i.e., LLS and ESQ (AMS-C)] as shown in **Table 2**.

**Table 2:** Oxygen saturation, acute mountain sickness and trail making tests during acute and acclimatization exposures.

Variables	*Cycle 1*				*Cycle 2*			
	Baseline SL	HA1	HA6	Recover SL	Baseline SL	HA1	HA6	Recover SL
SpO_2_ (%)	98.0 ± 0.9	80.2 ± 4.7*	83.7 ± 4.5*†	98.0 ± 1.2	98.4 ± 1.1	82.8 ± 6.6*	85.0 ± 4.0*	97.8 ± 1.3
Lake Louise Score (LLS)	0.8 ± 1.4	5.4 ± 3.1*	1.9 ± 2.0†	0.5 ± 0.8	1.7 ± 1.8	4.2 ± 2.7*	2.0 ± 2.1†	0.9 ± 1.2
AMS, *n* (%)	–	12 (57)	2 (10)	–	1 (5)	6 (29)	2 (10)	–
AMS-C (ESQ)	0.1 ± 0.2	1.0 ± 0.8*	0.2 ± 0.2†	0 ± 0.1	0.1 ± 0.2	0.6 ± 0.6*	0.2 ± 0.3†	0 ± 0
AMS, *n* (%)	–	13 (62)	0 (0)	–	1 (5)	6 (29)	2 (10)	–
Trail making test (TMT)								
Time (s)	49.0 ± 11.5	48.1 ± 13.3	42.4 ± 9.7*†	41.3 ± 8.8	41.5 ± 9.4	41.3 ± 9.6	37.7 ± 8.2*†	37.1 ± 7.9

*Values are means ± SD. SL, sea level; HA1, high altitude at day 1; HA6, high altitude day 6; n, numbers; SpO_*2*_, blood oxygen saturation; LLS, Lake Louise Score; AMS, acute mountain sickness; AMS-C, acute mountain sickness – cerebral; ESQ, Environmental Symptom Questionnaire. ^*a*^*n* = 20; ^∗^*p* < 0.05: Different from respective baseline SL; †*p* < 0.05: Different from respective HA1.*

**FIGURE 3:**
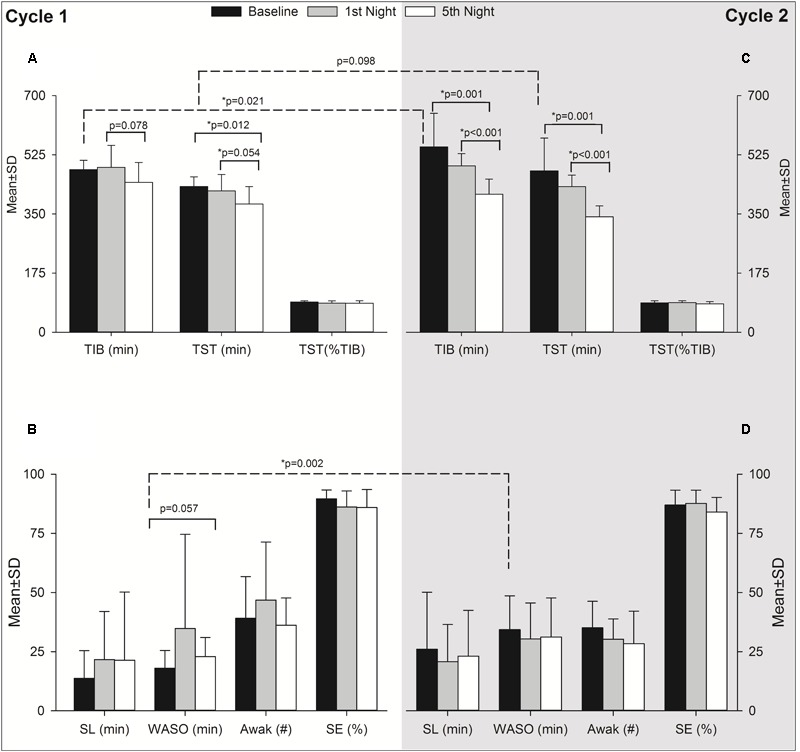
Effect of altitude on actigraphy sleep indices during acute and acclimatization exposure. It has two panels as *Cycle 1* (Left, **A**,**B**) and *Cycle 2* (Right, **C,D**). The *Y*-axis depicts sleep parameters with the mean ± standard deviation (Mean ± SD) at baseline, acute exposure (1st night altitude), and acclimatization night (5th night at altitude). The *X*-axis depicts different sleep parameters as reported from actigraphy during the first expedition of *Cycle 1* and *Cycle 2*. Black bars, baseline sleep at Santiago, Chile (520 m asl); gray bars, acute exposure to altitude, i.e., 1st night sleep at high altitude (2900 m asl) and empty bars, sleep after acclimatization at high altitude, i.e., 5th night sleep at high altitude in each cycle. The significance shown with dashed line indicates baseline comparisons of two cycles. The comparison with solid lines is between different time points of respective cycles. TIB, time in bed; TST, total sleep time; SL, sleep latency; WASO, wake after sleep onset; Awak, awakenings; SE, sleep efficiency; SD, standard deviation; min, minute; *p*, level of significance; *n*, number; %, percentage; ^∗^*p*, level of significance value with less than 0.05.

Actigraphy results are summarized in **Table 3**. Due to technical difficulties, data are given only up to the 5th night for 18 individuals in *Cycle 1* and 14 individuals in *Cycle 2* for the entire expedition (i.e., 9 nights including baseline, 7 nights at altitude and recovery) (**Table 3**). To explore the acute and acclimatization effects of altitude upon sleep indices, the baseline was compared with acute exposure (HA1) sleep and after acclimatization (HA6) sleep in each cycle as illustrated in **Figure 3**. In *Cycle 1*, the altitude had an adverse effect on total sleep time at the 5th night (i.e., after the acclimatization days). The individuals tended to spend less time in bed (*p* = 0.078, 1st vs. 5th nights) while wakefulness after sleep-onset time tended to increase (*p* = 0.057, baseline vs. 5th night) during the acclimatization night although they were not statistically significant (**Figures 3A and B**, *Cycle 1*). Interestingly, during *Cycle 2*, participants spent less time in bed (*p* = 0.001, baseline vs. 5th night; *p* < 0.001, 1st vs. 5th nights) and had decreased total sleep time (*p* = 0.001, 1st vs. 5th nights; *p* < 0.001, 1st vs. 5th nights) during prolonged exposure (5th night) compared to baseline and 1st night at altitude (**Figure 3C**). To find out the break point for the sleep disturbances observed during acclimatization exposure (5th night), we analyzed sleep indices of all the nights (N1, N2, N3, N4, and N5) with baseline (BL) using one-way repeated measures ANOVA (time^∗^6). The changes in total sleep time (TST) started on the 3rd night at altitude in both cycles – *Cycle 1* (BL: 431.3 ± 28.7 vs. N3: 369.7 ± 54.3, *p* = 0.008 and N1: 418.1 ± 48.6 vs. N3: 369.7 ± 54.3, *p* = 0.062) and *Cycle 2* (BL: 477.5 ± 96.9 vs. N3: 368.9 ± 61.5, *p* = 0.097 and N1: 430.9 ± 34 vs. N3: 368.9 ± 61.5, *p* = 0.021). The findings were further supported with a series of repeated measures ANOVA analyses (time^∗^3) as baseline (BL), acute exposure (N1, 1st night at altitude), and respective nights (N2, N3, N4, and N5). We observed significant changes in TST from 3rd night at altitude onward in both cycles – *Cycle 1* (BL: 431.3 ± 28.7 vs. N3: 369.7 ± 54.3, *p* = 0.002 and N1:418.1 ± 48.6 vs. N3: 369.7 ± 54.3, *p* = 0.0125) and *Cycle 2* (BL: 477.5 ± 96.9 vs. N3: 368.9 ± 61.5, *p* = 0.004 and N1:430.9 ± 34 vs. N3: 368.9 ± 61.5, *p* = 0.004).

**Table 3:** Actigraphy outcome of sleep parameters during expedition.

Variables	Santiago, 520 m	1st night	2nd night	3rd night	4th night	5th night	6th night	7th night	Santiago, 520 m
**Time in bed (min)**		
*Cycle 1*	481.4 ± 27.1	487.8 ± 65.4	511.2 ± 76.6	458.4 ± 47.6	451.7 ± 73.1	443.8 ± 58.3	–	–	–
*Cycle 2*	548.5 ± 99.2§	492.5 ± 35.9	506.9 ± 47.0	442.4 ± 70.7	463.2 ± 63.3	408.4 ± 44.7*†	507.0 ± 37.9	460.1 ± 57.1	534.5 ± 62.2
**Total sleep time (min)**		
*Cycle 1*	431.3 ± 28.7	418.1 ± 48.6	428.7 ± 90.5	369.7 ± 54.3	381.3 ± 72.7	379.7 ± 51.4*†	–	–	–
*TST (% of TIB)*	89.6 ± 3.7	86.1 ± 6.8	83.8 ± 11.8	80.8 ± 10.0	84.1 ± 4.7	85.9 ± 7.6	–	–	–
*Cycle 2*	477.5 ± 96.9	430.9 ± 34.0	409.7 ± 44.6	368.9 ± 61.5	391.1 ± 54.4	341.4 ± 32.2*†	415.9 ± 45.1	392.0 ± 60.8	439.7 ± 69.1
*TST (% of TIB)*	87.0 ± 6.3	87.6 ± 5.6	81.0 ± 6.7	83.6 ± 6.2	84.5 ± 4.0	83.9 ± 6.2	82.3 ± 9.4	85.1 ± 6.3	82.3 ± 8.7
**Latency (min)**		
*Cycle 1*	13.7 ± 11.7	21.6 ± 20.4	33.6 ± 40.1	25.6 ± 21.2	20.5 ± 19.4	21.4 ± 28.8	–	–	–
*Cycle 2*	26.0 ± 24.1	20.8 ± 15.7	41.6 ± 44.9	20.6 ± 21.9	21.5 ± 21.8	23.1 ± 19.3	41.9 ± 36.5	11.8 ± 15.1	48.4 ± 48.6
**Sleep efficiency (%)**		
*Cycle 1*	89.6 ± 3.7	86.1 ± 6.8	83.8 ± 11.8	80.9 ± 10.0	84.1 ± 4.6	85.9 ± 7.6	–	–	–
*Cycle 2*	87.0 ± 6.3	87.6 ± 5.6	81.0 ± 6.7	83.6 ± 6.2	84.6 ± 3.9	84.0 ± 6.2	82.3 ± 9.4	85.1 ± 6.2	82.3 ± 8.7
**Wake after sleep onset (min)**		
*Cycle 1*	18.0 ± 7.5	34.8 ± 39.8	26.7 ± 16.7	38.2 ± 43.2	23.5 ± 9.4	22.9 ± 8.1	–	–	–
*Cycle 2*	34.3 ± 14.3^§^	30.4 ± 15.2	32.7 ± 17.4	33.6 ± 21.5	30.3 ± 15.3	31.2 ± 16.5	25.1 ± 10.7	30.8 ± 12.4	31.9 ± 14.7
**Awakenings, #**		
*Cycle 1*	39.1 ± 17.6	46.8 ± 24.5	38.4 ± 16.7	50.8 ± 34.0	39.7 ± 16.2	36.2 ± 11.5	–	–	–
*Cycle 2*	35.1 ± 11.2	30.2 ± 8.6	37.1 ± 14.9	33.8 ± 17.4	30.2 ± 10.5	28.4 ± 13.8	26.8 ± 9.8	31.7 ± 11.3	33.4 ± 12.1

*Sleep indices from actigraphic measurements. Values are means ± SD. m, meter; min, minute; ^#^: Awakenings. *Cycle 1* had 18 subjects and *Cycle 2* had 14 subjects with complete actigraphy data due to field constraints and device issues. ^∗^*p* < 0.05: Different from respective baseline SL. †*p* < 0.05: Different from 1st night at high altitude. ^§^*p* < 0.05: Different from BL of *Cycle 1*.*

**FIGURE 4:**
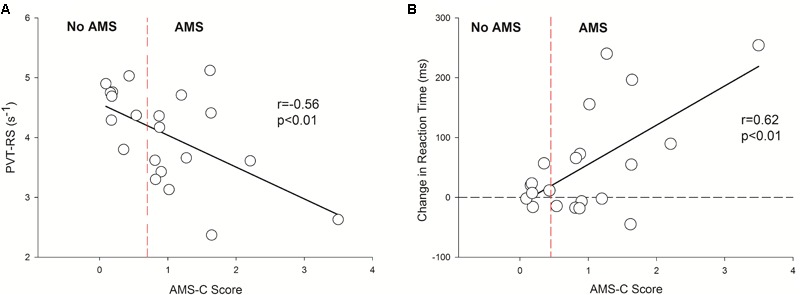
Inverse correlation between reaction speed vs. AMS-C score and positive association with changes in reaction time (HA1 – BL) vs. AMS-C score. It has two panels, left panel **(A)** shows negative correlation between PVT-RS vs. AMS score and the right panel **(B)** shows positive correlation between changes in reaction time (HA1 – BL) vs. AMS score. The *Y*-axis depicts reaction speed during the first exposure at altitude in **A** and the changes in reaction times (HA1, reaction time of high altitude at Day 1 – BL, reaction time at baseline) in **B** during acute exposure at altitude during *Cycle 1*. In both panels, the *X*-axis depicts AMS-C score during the acute exposure *Cycle 1*. The vertical dashed red lines in both panels separates no AMS subjects from AMS with the cut-off of a ≥ 0.7 AMS-C score. In **B**, the horizontal dashed line passing through zero of *Y*-axis is a reference line separating negative (i.e., subjects with decreased reaction time at high altitude) and positive (i.e., subjects with increased reaction time at high altitude) values of changes in reaction times. PVT-RS, psychomotor vigilance test reaction speed; AMS, acute mountain sickness; AMS-C, acute mountain sickness – cerebral; BL, baseline at low altitude; HA1, acute high altitude exposure; s, seconds; ms, milliseconds; *r*, Pearson’s correlation; *p*, level of significance.

Finally, the acute exposure to HA is associated with increased incidence of AMS which wears off with acclimatization (**Table 2**). The incidence of AMS decreased by > 50% during *Cycle 2* (HA1) exposure compared to *Cycle 1* (HA1). Next, the AMS-C score was negatively correlated (*r* = -0.56, *p* < 0.01) with PVT-RS. Altitude adversely affected PVT-RS during acute exposure and was associated with increased AMS-C scores as illustrated in **Figure 4A**. The mean reaction time changes (mean reaction at altitude – mean reaction time at baseline) were positively correlated (*r* = 0.62, *p* < 0.01) with AMS-C scores i.e., higher AMS-C scoring individuals took a longer time to react at HA during acute exposure as shown in **Figure 4B**. The individuals recovered during the acclimatization phase (i.e., there was no such correlation among these parameters); this recovery was sustained during *Cycle 2* even after 1 week of rest at LA. Multiple regression analyses revealed that AMS-C score (*p* = 0.01) but not the total sleep time and blood oxygen saturation was the independent predictor of PVT-RS during acute HA exposure (HA1) of *Cycle 1* (**Table 4**). However, the association was abolished during acclimatization (HA6) and re-exposures (*Cycle 2*). The clinical outcome measures were returned to the baseline during recovery period, i.e., after returning to sea level.

**Table 4:** Multiple regression analyses of the relationships between PVT-RS with total sleep time, AMS-C score and SpO_2_ during acute and acclimatization exposures.

Predictors	*Cycle 1*										*Cycle 2*									
	HA1					HA6					HA1					HA6				
	B	SE	β	*p*-value	95% CI	B	SE	β	*p*-value	95% CI	B	SE	β	*p*-value	95% CI	B	SE	β	*p*-value	95% CI
Intercept	6.556	3.51	–	0.08	-0.974, 14.086	2.263	4.340	–	0.610	-7.045, 11.571	1.502	4.105	–	0.722	-7.645, 10.649	2.314	4.303	–	0.603	-7.274, 11.901
TST (min)	-0.004	0	-0.260	0.25	-0.012, 0.003	0.002	0.004	0.116	0.659	-0.007, 0.010	0.003	0.007	0.159	0.614	-0.011, 0.018	-0.001	0.006	-0.050	0.877	-0.013, 0.012
AMS-C Score^∗^	-0.595	0.21	-0.610	0.01	-1.049, -0.140	1.062	1.208	0.256	0.394	-1.529, 3.653	-0.394	0.343	-0.348	0.278	-1.157, 0.370	0.739	1.273	0.173	0.575	-2.098, 3.575
SpO_2_ (%)	-0.001	0.04	0.005	0.98	-0.080, 0.078	0.017	0.049	0.098	0.739	-0.089, 0.123	0.024	0.030	0.232	0.456	-0.044, 0.091	0.032	0.039	0.263	0.423	-0.054, 0.118

*Psychomotor vigilance test reaction speed (PVT-RS) was used as dependent variable while total, sleep time, AMS-C score and SpO_*2*_ were used as predictor variables. The level of significance was considered at *p* < 0.05. B, unstandardized regression coefficients; SE, standard error of the coefficient; β, standardized coefficients; 95% CI, 95% confidence interval; TST, total sleep time; AMS-C score, acute mountain sickness – cerebral score; SpO_*2*_, blood oxygen saturation.*

## Discussion

### Major Findings

Several novel findings emerged from this study. First, the PVT-RS was reduced with acute exposure to HA and normalized with acclimatization over 6 days. Further, acclimatization effects were retained upon re-exposure to altitude even after spending a week at sea level. Second, there were 62% and 29% of individuals suffering from AMS in *Cycle 1* and *Cycle 2*, respectively, with an immediate HA exposure. However, after 6 days of acclimatization, the incidence of AMS was significantly reduced to none and 10% in *Cycle 1* and *Cycle 2*, respectively. The incidence of AMS, during acute exposures (HA1), was decreased by more than 50% in *Cycle 2* compared to *Cycle 1*. Third, total estimated sleep time were decreased during *Cycle 2*. Fourth, the AMS score was associated with worsened PVT performance (decrease in reaction speed and increase in mean reaction time). This appears to be unrelated to estimated sleep duration and blood oxygen saturation. The altitude-naive individuals recovered upon descending to sea level after each cycle.

### The Psychomotor Vigilance Test: Daytime Alertness/Sustained Attention

Sleep-disordered breathing and intermittent hypoxia are associated with excessive daytime sleepiness, loss of focus, memory impairment, and difficulty with tasks that demand sustained attention (Morrell and Twigg, 2006; Dempsey et al., 2010; Beaudin et al., 2017a,b). The PVT, a validated neurocognitive assay for sustained vigilance attention (Dorian et al., 2005; Lim and Dinges, 2008), is impaired in patients with sleep-disordered breathing (Sforza et al., 2004; Kim et al., 2007), sleep-deprived individuals (Lim and Dinges, 2008; Basner and Dinges, 2011), circadian rhythm mismatch (Van Dongen and Dinges, 2005), and in professionals whose work demands continuous focus in challenging operational environments such as aviation pilots, field engineers, and military personnel (Gander et al., 2014, 2016; Shattuck and Matsangas, 2015; Song et al., 2017). The PVT reaction speed, lapses, mean, and median reaction times as well as fastest and slowest 10% reaction times are particularly sensitive in assessing alertness among sleep-deprived individuals (Dorian et al., 2005; Basner and Dinges, 2011). The PVT has also been tested at HA among healthy individuals, in whom no changes in PVT parameters were noted despite a considerable number of periodic breathing episodes and sleep disturbances (Latshang et al., 2013). Seemingly, contrary to this finding, we report impairments in mean/median reaction times, reaction speed, and reciprocal of slowest 10% reaction time during acute exposure (**Figure 2**) while sleeping at moderate altitude and traveling to very HA during the day. The discrepancy in these PVT outcomes might be due to the magnitude and duration of altitude exposure (i.e., overall study design difference). In the previous study (Latshang et al., 2013), the highest altitude gained was 2590 m asl whereas the sleeping altitude in our expedition was 2900 m asl and study participants were exposed to 5050 m asl daily for about 6–8 h per day. The PVT-RS during acute exposure was lower at ALMA (4.1 s^-1^) vs. 2590 m (5.0 s^-1^). The PVT changes improved after acclimatization over 6 days – the acclimatization effect was retained during *Cycle 2* exposure after spending a week at sea level (except for the reciprocal of slowest 10% reaction time that followed a similar pattern as in *Cycle 1*) (**Figure 2**). The TMT showed improvement over time at altitude, which might be due to learning effects with acclimatization (Pagani et al., 1998; Buck et al., 2008).

### Sleep at Altitude: Sleep Indices From Actigraphy

We assessed sleep with wrist actigraphy (Sadeh, 2005) which has also been used at HA, having been validated against the gold standard technique of polysomnography (Latshang et al., 2016). The sleep indices from actigraphy in *Cycle 1* (baseline and subsequent five nights at altitude) and *Cycle 2* (baseline, seven nights at altitude, and recovery at sea level) were not significantly different across all time points (**Table 3**). However, on comparing three time points (i.e., baseline, acute, and acclimatization exposure), the total sleep time was impaired during the 5th night at altitude compared to the baseline and 1st night at altitude in *Cycle 1*. Similarly, the time in bed tended to decrease on the 5th night compared to the 1st night, and wakefulness after sleep onset tended to increase on the 5th night compared to baseline (**Figure 3**). During *Cycle 2*, we found both time in bed and total sleep time were decreased on the 5th night compared to baseline and 1st night at altitude (**Figure 3**). It is noteworthy that in both cycles, we observed impaired total estimated sleep time (actigraphy), which is in agreement with previous reports of polysomnography parameters at altitude (Latshang et al., 2016). The sleep disturbance as reflected in TST appears to have significantly affected from 3rd night onward. The inflexion point of sleep disturbance may be related to the time domains of hypoxic ventilatory acclimatization at HA (Pamenter and Powell, 2016). Worsening sleep indices with acclimatization are in contrast to the improvements observed in PVT performances and blood oxygen saturation. The findings on sleep indices are consistent with Latshang et al. (2013) who found disturbance in nocturnal breathing and sleep during first four nights of altitude stay although they did not find similar changes in PVT performance. The PVT performance seems to be influenced by improvements in AMS symptoms and blood oxygen saturation rather than sleep disturbance. The decreased total sleep time index might be related to HA periodic breathing, which is a consequence of an exaggerated hypoxic ventilatory response (Lahiri et al., 1983). Alternatively, individuals might have been sleep deprived and exhausted in the 1st nights after the transfer from sea level and therefore might have required recovery sleep time.

### Acute Mountain Sickness and Blood Oxygen Saturation

We found very high AMS incidence (62%) with acute altitude exposure, which decreased with acclimatization (none) in *Cycle 1*, and the acclimatization benefit was observed in *Cycle 2* as well (29% vs. 10%). The acclimatization gained in *Cycle 1* reduced AMS incidence by > 50% during acute exposure of *Cycle 2*. The findings are consistent with previous studies that acclimatization reduces AMS incidence and severity but not entirely protective (Beidleman et al., 2004; Jackson et al., 2010; Muza et al., 2010; Schommer et al., 2010). We used both the LLS (Roach et al., 1993) and the AMS-C (Sampson et al., 1983) scores for the diagnosis of AMS (Maggiorini et al., 1998; Dellasanta et al., 2007), and they were in agreement with each other in most instances (**Table 2**). Similar to Nussbaumer-Ochsner et al. (2012), we found improved oxygen saturation after acclimatization, but we did not observe an improvement in sleep indices. The acute and acclimatization exposures influenced arterial blood pressures in both cycles compared to their respective baselines (**Table 1**). The peak HG strength changed during altitude exposures returned to closer to the baseline during acclimatization exposures (**Table 1**).

Higher AMS-C scores (i.e., more severe symptoms of AMS) were negatively correlated with the PVT-RS (**Figure 4A**) during acute exposure of *Cycle 1*. Hence, AMS seems to adversely affect alertness and performances that require sustained attention. Similarly, increasing reaction time (i.e., increased the change in reaction time) was positively correlated with the AMS-C score (**Figure 4B**); individuals who had higher AMS scores took longer to react to PVT stimuli. The findings suggest that the individuals suffering from AMS might struggle to perform delicate work tasks such as maintenance, scientific observations, and reporting, engineering and operation of heavy-duty equipment. Furthermore, multiple regression analysis revealed that except AMS-C score, the estimated sleep duration and blood oxygen saturation are not associated with PVT-RS (**Table 4**). The findings further support the previous evidence that hypoxia resulting from HA has global cerebral effects (Kramer et al., 1993; Wilson et al., 2009; Maa, 2010; Roach et al., 2014). The associations were abolished after acclimatization (HA6, i.e., during HA exposure at Day 6). Further, the acclimatization obtained in *Cycle 1* was carried over during *Cycle 2* even after 1 week of LA rest. These findings support the practice followed by the Chilean HA mining workers (Richalet et al., 2002; Farias et al., 2006). The assessment of AMS along with the incorporation of PVT could provide insight into the status of HA illness and its impact on alertness/fatigue and safety margins during HA work (Vearrier and Greenberg, 2011; Abe et al., 2014).

### Strengths and Limitations

This study was designed to replicate a typical work schedule and ascent profile of the employees at the ALMA Observatory (Chile) with two working cycles separated by a week of rest at LA. The 1-week break at LA is an attempt to minimize the ill effects of long-term sustained chronic hypobaric hypoxia (Richalet et al., 2002; Vearrier and Greenberg, 2011) and at the same time return to work while the acclimatization effects acquired in the previous exposure(s) are still retained (Farias et al., 2006; Muza et al., 2010). However, the extent to which this exposure profile is effective to optimize acclimatization while mitigating AMS symptoms has been explored for the first time. Hence, this study provides insight into the workers’ schedule in relation to optimum schedule to maximize their work output while increasing work safety and reducing health risks. Therefore, the results from this study have implications in reducing health hazards and improving work performance for individuals engaging in HA activities that require high daytime attentiveness such as astronomical observation, engineering, computing, scientific reporting, and operation of heavy-duty equipment.

Our study has a few limitations. First, our sample contains a relatively small group of 21 active young individuals. Due to logistical reasons, the expedition was designed only for two regular work cycles. Workers at the ALMA Observatory are older (ALMA workers’ average age is between 35 and 40 years), and they have been working for longer durations (i.e., repeated cycles of HA exposure throughout the year). Second, we could only retrieve complete actigraphy data sets from 18 individuals during *Cycle 1* (and up to 5 nights) and 14 individuals during *Cycle 2* (complete cycle). Finally, we had relatively heterogeneous population with the mixture of nationalities (Canadians and Swiss) and their background of altitude exposure (Calgary, 1100 m asl; Zurich, 490 m asl).

## Conclusion

The present study reports novel findings from repeated exposures to very HA with sleep at HA interspersed with a week of break at low attitude. With acute exposure to HA, individuals suffered from decreased PVT performances, hypoxemia, and increased incidence of AMS. After acclimatization, the individuals revealed slightly less hypoxemia, a lower incidence of AMS and had improved sustained attention functions. The acclimatization effect acquired over a week of HA exposure was retained on a subsequent cycle of exposure even after spending 1 week at near sea level. The individuals with higher AMS-C scores had impaired psychomotor reaction speeds. The sleep indices, especially total estimated sleep time, were worsened during re-exposure, showing no influence of acclimatization on sleep, which is similar to the previous observations that sleep disturbances such as periodic breathing continue to occur (Bloch et al., 2010) despite improved clinical outcome parameters and PVT performances.

## Author Contributions

MJP, KB, SU, JR, PB, ML, LM, AD, MF, and SH were involved in the study design, data collection, analysis, manuscript preparation, and submission. MP was involved in the data analysis and took lead in the literature review, manuscript preparation, and submission process.

## Conflict of Interest Statement

The authors declare that the research was conducted in the absence of any commercial or financial relationships that could be construed as a potential conflict of interest.
